# Gravity Waves and Wind-Farm Efficiency in Neutral and Stable Conditions

**DOI:** 10.1007/s10546-017-0307-5

**Published:** 2017-10-13

**Authors:** Dries Allaerts, Johan Meyers

**Affiliations:** Department of Mechanical Engineering, KU Leuven, Celestijnenlaan 300, Box 2421, 3001 Leuven, Belgium

**Keywords:** Atmospheric gravity waves, Boundary-layer stability, Large-eddy simulation, Low-level jet, Wind farm

## Abstract

We use large-eddy simulations (LES) to investigate the impact of stable stratification on gravity-wave excitation and energy extraction in a large wind farm. To this end, the development of an equilibrium conventionally neutral boundary layer into a stable boundary layer over a period of 8 h is considered, using two different cooling rates. We find that turbulence decay has considerable influence on the energy extraction at the beginning of the boundary-layer transition, but afterwards, energy extraction is dominated by geometrical and jet effects induced by an inertial oscillation. It is further shown that the inertial oscillation enhances gravity-wave excitation. By comparing LES results with a simple one-dimensional model, we show that this is related to an interplay between wind-farm drag, variations in the Froude number and the dispersive effects of vertically-propagating gravity waves. We further find that the pressure gradients induced by gravity waves lead to significant upstream flow deceleration, reducing the average turbine output compared to a turbine in isolated operation. This leads us to the definition of a non-local wind-farm efficiency, next to a more standard wind-farm wake efficiency, and we show that both can be of the same order of magnitude. Finally, an energy flux analysis is performed to further elucidate the effect of gravity waves on the flow in the wind farm.

## Introduction

It is well-known that the superposition of turbine wakes in a wind farm reduces the wind speed and increases the turbulence intensity inside and above the farm, and in past years, many studies have focused on simulations and measurements of wake interactions in wind farms (see, e.g., Frandsen et al. 2006; Barthelmie et al. 2009; Calaf et al. 2010; Wu and Porté-Agel 2013). Recently, Allaerts and Meyers (2017) showed using large-eddy simulations (LES) that in some situations large wind farms interact with the atmosphere on a much larger scale by exciting atmospheric gravity waves. Earlier, Smith (2010) considered this interaction and the impact on the wake efficiency with a simplified linear model. Wind-farm induced gravity waves are triggered by the upward displacement of the boundary-layer top due to flow blockage inside the farm and they are physically similar to the waves excited by flow over hills and mountain ranges. Jiang and Doyle (2008) found that diurnal heating and cooling at the surface has a profound impact on the strength of mountain waves. While unstable stratification of the boundary layer weakens the mountain waves and reduces the surface drag by up to 90%, stable stratification may increase the drag several times compared to the hydrostatic wave drag without boundary-layer effects. Several other studies also found enhanced gravity-wave activity during nighttime when the surface heat flux was negative (Brinkmann 1974; Whiteman and Whiteman 1974; Doyle et al. 2005; Valkonen et al. 2010). In large wind farms, gravity waves impose significant pressure gradients in the boundary layer and play an important role in redistributing energy throughout the farm (Allaerts and Meyers 2017). The objective of the current study is to investigate the impact of stable stratification on wind-farm performance and boundary-layer flow, with a particular interest in gravity waves.

Over the past few years, a considerable amount of literature has been published on the interaction between wind farms and the atmospheric boundary layer (ABL); see Stevens and Meneveau (2017) for a recent review. Many of these studies use large-eddy simulations in conjunction with field measurements and wind-tunnel experiments to understand the complex flow behaviour. The number of wind-farm–LES studies considering stable conditions, however, remains rather limited because of the numerical challenges associated with stably-stratified boundary layers (SBLs). Lu and Porté-Agel (2011) were the first to study the effect of stable stratification on wind farms. Their LES modelled the asymptotic limit of an “infinite” wind farm using a minimal flow unit approach containing one turbine. The atmospheric conditions were thereby set to match those of the first Global Energy and Water Cycle Experiment (GEWEX) Atmospheric Boundary Layer Study (GABLS) (Beare et al. 2006). Further, Witha et al. (2014) and Dörenkämper et al. (2015) analysed the impact of stability on the German wind farm EnBW Baltic 1, which consists of 21 wind turbines and lies 16 km offshore in the southern Baltic Sea. Stable conditions were achieved by either prescribing a constant negative surface heat flux or by advecting warm air masses over a cool surface with low surface roughness. The latter approach corresponds to a land–sea transition and gives rise to a stable internal boundary layer, a flow regime that occurs frequently in the Baltic Sea (see, e.g., Tjernström and Smedman 1993; Smedman et al. 1997; Lange et al. 2004). More recently, Abkar et al. (2016) investigated the wake flow in an idealised finite-size wind farm with 36 turbines over the course of a full diurnal cycle. Cortina et al. (2016) and Sharma et al. (2017) investigated the flow behaviour in an infinite wind-turbine array during a diurnal cycle with forcing conditions extracted from the Cooperative Atmosphere-Surface Exchange Study 1999 (CASES-99) field campaign (Poulos et al. 2002).

The aforementioned wind-farm–SBL studies all consider either fairly small wind farms (i.e., at most $6\times 6$ wind turbines) or the asymptotic limit of infinitely large wind farms. Accordingly, the conclusions of these studies only focus on local effects such as wind-turbine wake structure and power loss, and none of these studies considered effects on a larger scale such as gravity waves. In the current work, we perform large-eddy simulations of a large wind farm consisting of 126 wind turbines arranged in an aligned pattern (with respect to the initial flow direction), covering an extensive surface area of $10\;\mathrm {km}\times 4.8\;\mathrm {km}$. Moreover, we simulate the stable boundary layer on a numerical domain size that is much larger than earlier studies, allowing us to study the impact of surface-layer stability on wind-farm induced gravity waves. We show that wind-energy extraction not only affects the region downstream of the farm, but also influences the wind speed and direction upstream of the farm considerably.

The simulations performed in this work consider an equilibrium, onshore, conventionally neutral boundary layer (CNBL) developing into a stable boundary layer due to a prescribed surface cooling rate. This situation represents part of the diurnal cycle, similar to a late-afternoon ABL transition into a nocturnal boundary layer. Two cases with surface cooling rates of 0.25 and $0.75\;\mathrm {K}\,\mathrm {h}^{-1}$ are simulated to yield varying surface-layer stability. The adopted simulation strategy allows us to start from a fully developed turbulent boundary-layer regime with well-defined atmospheric conditions such as boundary-layer height and wind direction.

This paper is further organised as follows. The numerical set-up of the wind farm and the computational domain is summarised in Sect. 2. Next, Sect. 3 describes the initialisation and transition of the boundary-layer flow in the precursor simulation. The wind-farm performance and boundary-layer flow is discussed in Sect. 4, and the influence of atmospheric gravity waves is investigated in Sect. 5. A further analysis of the energy fluxes through the boundary layer and the wind farm is presented in Sect. 6, and conclusions are given in Sect. 7.

## Numerical Aspects

We perform large-eddy simulations of a large, onshore wind farm subject to varying surface cooling rates. The simulations are performed with SP-Wind, an in-house research code developed over the last 10 years (for details, see among others, Meyers and Sagaut 2007; Meyers and Meneveau 2010; Allaerts and Meyers 2015; Munters et al. 2016). SP-Wind uses pseudo-spectral discretisation schemes in the horizontal directions, and a fourth-order energy-conserving finite difference scheme in the vertical direction (Verstappen and Veldman 2003). Time integration is performed using a classic four-stage fourth-order Runge–Kutta scheme, with a timestep based on a Courant–Friedrichs–Lewy number of 0.4. The subgrid-scale motions are modelled with the turbulent kinetic energy (TKE) model developed by Deardorff (1980), and classic Monin–Obukhov similarity theory is used to specify the boundary conditions near the surface. Following Allaerts and Meyers (2017), wind-farm entrance effects and boundary-layer development are included by using the concurrent-precursor method (Stevens et al. 2014; Munters et al. 2016), extended to include the effects of thermal stratification and Coriolis forces. Details of the LES methodology can be found in Appendix 1.

**Fig. 1:**
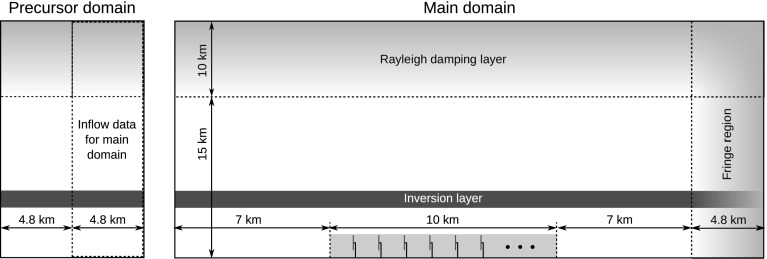
Sketch of the computational set-up in the precursor and main domain (*x*–*z* cut of the three-dimensional domain), showing the relative positions of the wind farm, the fringe region and Rayleigh damping layer. The vertical scale is exaggerated as the inversion layer height is about 1 km

The set-up of the wind farm and the numerical domain is to a large extent inspired by the simulations of Allaerts and Meyers (2017). A summary and outline of the computational set-up are given in Table 1 and Fig. 1. The wind farm consists of 126 generic turbines with a diameter $D=100\;\mathrm {m}$ and a hub height $z_\mathrm{h}=100\;\mathrm {m}$. The turbines are represented by an Actuator Disk Model (ADM) with a disk-based thrust coefficient $C_\mathrm{T}^\prime =4/3$, similar to earlier work by Calaf et al. (2010), Meyers and Meneveau (2010) and Goit and Meyers (2015). Further details on the actuator disk methodology are found in Appendix 1. In view of the high computational cost per simulation, our study considers only one wind-farm layout in which the turbines are arranged in an aligned pattern of 14 rows by 9 columns with respect to the mean flow direction in conventionally neutral conditions (i.e., before the transition to stable conditions starts). With a turbine spacing of $s_xD=7.5D$ and $s_yD=5.33D$, the wind farm covers an area of roughly $10\;\mathrm {km}\times 4.8\;\mathrm {km}$. We have chosen this size to be slightly smaller than the wind farm studied by Allaerts and Meyers (2017) in order to constrain the computational cost given the finer resolution required for stable boundary-layer simulations. Note that the amplitude of gravity waves is expected to depend on the flow blockage and therefore on the size of the wind farm. However, in the current study, we do not investigate different wind farm sizes, and this dependence may be an interesting topic for future research.

**Table 1:** Set-up of the computational domain and wind farm

Main domain size	$L_x\times L_y\times L_z=28.8\;\mathrm {km}\times 4.8\;\mathrm {km} \times 25\;\mathrm {km}$
Precursor domain size	$L_x\times L_y\times L_z=9.6\;\mathrm {km}\times 4.8\;\mathrm {km} \times 25\;\mathrm {km}$
Main domain grid	$N_x\times N_y\times N_z=2304\times 384\times 700$
Precursor domain grid	$N_x\times N_y\times N_z=768\times 384\times 700$
Vertical grid	$$\left\{ \begin{array}{llc} N_{z,1}=300, &{} \varDelta z = 5\;\mathrm {m}, &{} 0<z<1.5\;\mathrm {km} \\ N_{z,2}=350, &{} \varDelta z = 5{-}40\;\mathrm {m}, &{} 1.5<z<15\;\mathrm {km}\\ N_{z,3}=50, &{} \varDelta z = 40{-}300\;\mathrm {m}, &{} 15<z<25\;\mathrm {km} \end{array} \right. $$
Horizontal grid resolution	$\varDelta x = \varDelta y= 12.5\;\mathrm {m}$
Turbine arrangement	$14\;\mathrm {rows}\times 9\;\mathrm {columns}$
Turbine dimensions	$D=100\;\mathrm {m}\quad \mathrm {and} \quad z_\mathrm{h}=100\;\mathrm {m}$
Turbine spacing	$s_x = 7.5D \quad \mathrm {and} \quad s_y = 5.33D$

Atmospheric turbulence is generated in a precursor domain with horizontal domain size equal to $L_x\times L_y=9.6\;\mathrm {km}\times 4.8\;\mathrm {km}$ (see Fig. 1). The main domain is three times longer than the precursor (i.e., $L_x=28.8\;\mathrm {km}$) in order to allow the investigation of non-local wind-farm effects. Moreover, the main domain contains a fringe region in the *x*-direction that serves two purposes. First, the fringe region is used to force the flow to the desired inflow conditions obtained in the precursor domain. Second, outward propagating gravity waves are absorbed by the fringe region in order to avoid numerical reflections at the domain boundaries. Using the simplified 2D potential flow solver developed by Allaerts (2016, Appendix C), the configuration of the fringe region is optimized to minimize reflections and to yield correct predictions for boundary layer and pressure perturbation fields (see also discussion in Sect. 5.1). The optimal parameters are a length of 4.8 km and a damping coefficient of $0.03\;\mathrm {s}^{-1}$. A horizontal grid resolution of $\varDelta x=\varDelta y=12.5$ m was found to provide sufficiently accurate results at an affordable computational cost (see Allaerts (2016) for a detailed grid sensitivity and validation study). The spatial layout of the main domain is shown in Fig. 1 and is very similar to that used by Allaerts and Meyers (2017) (cf. their Fig. 2): The wind farm is placed in the middle of the domain and is separated by 7 km in both the upstream and downstream directions from the fringe region.

The numerical domain is periodic in the spanwise direction and the wind farm spans the full width of the domain, which means that the current set-up simulates the asymptotic limit of an “infinitely” wide wind farm. Thus, compared to real wind farms of finite width, our simulations will overpredict wind-farm flow blockage and gravity-wave excitation as the wind cannot flow around the farm in a spanwise direction. Consequently, the results of this study roughly reflect the flow behaviour in the middle of a large wind farm with a width that exceeds the boundary-layer height by over an order of magnitude. The numerical domain is sufficiently wide so that streamwise elongated turbulent structures occurring in neutral conditions are advected through the domain before they are recycled via the spanwise periodic boundary conditions. The mean flow direction changes in stable conditions, but this is not a problem as the size of the turbulent structures also decreases drastically in this case.

The vertical domain size and vertical grid resolution are equal to that of case S1 of Allaerts and Meyers (2017), i.e., a vertical grid resolution of 5 m up to a height of 1.5 km (300 grid points), above which the grid is stretched in two steps: a resolution of 5–40 m up to $z=15\;\mathrm {km}$ (350 grid points and grid stretching factor $f_\mathrm{s}=1.0966$) and a resolution of 40–300 m above 15 km (50 grid points and $f_\mathrm{s}=1.0689$). Rayleigh damping is applied in the region above 15 km with a damping coefficient of $0.0001\;\mathrm {s}^{-1}$ to alleviate gravity-wave reflections [following the method of Taylor and Sarkar (2007), we estimate the wave reflection at the upper boundary to vary between 8 and 16% in terms of vertical kinetic energy, which is somewhat higher than the wave reflection in Allaerts and Meyers (2017)]. Note that the Boussinesq approximation still applies despite the large vertical extent of the domain as the characteristic vertical displacement (on the order of 100 m in our simulations) remains small compared to the density scale height of the atmosphere, which is typically on the order of 10 km (Wyngaard 2010).

## Precursor Simulation: CNBL to SBL Transition

In the precursor domain, the evening transition from a daytime CNBL to a surface-cooled SBL at night is simulated in order to provide inflow conditions for the wind farm in the main domain. First, an equilibrium CNBL is simulated, the set-up and result of which are discussed in Sect. 3.1. Subsequently, surface cooling is activated and the CNBL evolves into an SBL, which is described in Sect. 3.2.

### An Equilibrium Onshore CNBL

The spin-up of the equilibrium CNBL is comparable to the procedure used by Allaerts and Meyers (2017). We use similar atmospheric conditions, i.e., barotropic conditions with a geostrophic wind speed of $G=12\;\mathrm {m}\,\mathrm {s}^{-1}$ and Coriolis parameter $f_\mathrm{c}=10^{-4}\;\mathrm {s}^{-1}$, and a free atmosphere lapse rate $\partial \theta / \partial z = 1\;\mathrm {K}\,\text {km}^{-1}$, yielding a Brunt–Väisälä frequency $N=[(g/\theta _0) \,\partial \theta /\partial z]^{1/2}$ equal to $0.0058\;\mathrm {s}^{-1}$. Further, the height of the inversion layer is chosen to be 1000 m and the mixing layer temperature $\theta _\mathrm{m}$ is $15^\circ \;\mathrm {C}$, which is also used as the reference temperature $\theta _0$. The surface roughness length $z_0$ is set to 0.1 m to represent flow over land.

We choose the strength of the inversion layer to be high enough to limit turbulent entrainment and to keep the CNBL in equilibrium. An estimate can be obtained with the empirical formulation of Csanady (1974) for the asymptotic depth *h* of the CNBL,

1 $$\begin{aligned} h=A\frac{\theta _0}{g\varDelta \theta }u_*^2, \end{aligned}$$

with $u_*$ the friction velocity, $\varDelta \theta$ the inversion strength and $A\approx 500$ an empirical constant (Csanady 1974; Tjernström and Smedman 1993). An estimate of the friction velocity can be obtained from the CNBL model developed by Allaerts and Meyers (2015). Solving the implicit equation for the geostrophic drag $C_\mathrm{g}=u_*/G$ (i.e., their Eq. 35) with the given surface roughness, boundary-layer height and Rossby number yields a friction velocity of $0.53\;\mathrm {m}\,\mathrm {s}^{-1}$. Equation 1 then yields $\varDelta \theta >4.06\;\mathrm {K}$, so we set the inversion strength to 5 K.

Initial velocity and potential-temperature profiles are obtained with the approach of Allaerts and Meyers (2015), and random divergence-free perturbations with an amplitude of 0.1*G* are added in the velocity field below 100 m to trigger turbulence. Only the lowest 5 km of the numerical domain is simulated as no large-scale gravity waves occur during the first spin-up, and Rayleigh damping is applied between 2 and 5 km. Furthermore, the wind-angle controller of Allaerts and Meyers (2015) is activated during this step to align the flow direction at hub height with the *x*-direction. The CNBL is allowed to develop for 15 h in the precursor domain until a quasi-steady, fully turbulent state is reached.

Some steady-state parameters of the equilibrium state are given in Table 2 and compared with case S1 of Allaerts and Meyers (2017), which has a much lower surface roughness of $z_0=2\times 10^{-4}\;\mathrm {m}$. After 15 h, the height of the inversion centre is almost unchanged and the boundary-layer growth is very small, demonstrating that the CNBL is in equilibrium and that an adequate inversion strength was chosen. The higher surface roughness in the current simulations leads to lower wind speeds and higher turbulence intensities at hub height. The friction velocity is also higher, and matches very well with our initial estimate. Further, the lower wind speeds near the surface cause the wind to turn more towards the direction of the pressure gradient, which leads to a larger geostrophic angle compared to the lower surface roughness case of Allaerts and Meyers (2017).

**Table 2:** Steady state parameters of the onshore equilibrium CNBL, including the height of the inversion-layer centre $h_1$, the boundary-layer growth $\partial h_1/\partial t$, the hub-height velocity $M_\mathrm{hub}$, the friction velocity $u_*$, the geostrophic wind angle $\alpha$ and the turbulent intensity at hub height $\hbox {TI}=(\langle \overline{u_i^\prime u_i^\prime }\rangle /3)^{1/2}/M_\mathrm{hub}$

	$h_1$ (m)	$\partial h_1/\partial t \;(\mathrm {mm}\,\mathrm {s}^{-1})$	$M_\mathrm{hub}\;(\mathrm {m}\,\mathrm {s}^{-1})$	$u_*\;(\mathrm {m}\,\mathrm {s}^{-1})$	$\alpha \;(^\circ )$	TI (%)
$z_{0}=0.1$ m	1061	0.30	9.54	0.515	$-$17.35	8.35
$z_0=0.0002$ m	1058	0.13	10.96	0.310	$-$7.72	4.18

The values for a lower surface roughness are given for reference and correspond to case S1 of Allaerts and Meyers (2017) (see their Table 2)

After the spin-up, the simulation is advanced in time for an additional 20 min during which both main and precursor domain are simulated. During this period, which corresponds to about one wind-farm flow-through time, the wind farm goes through its start-up phase, the turbine yaw controllers converge to a steady state and the flow in the main domain adapts to the presence of the wind turbines. When all transitional effects of the wind-farm start-up have died out, the wind-angle controller in the precursor domain is turned off and simulations of the evening transition can start. This time is defined as $t=0$.

**Fig. 2:**
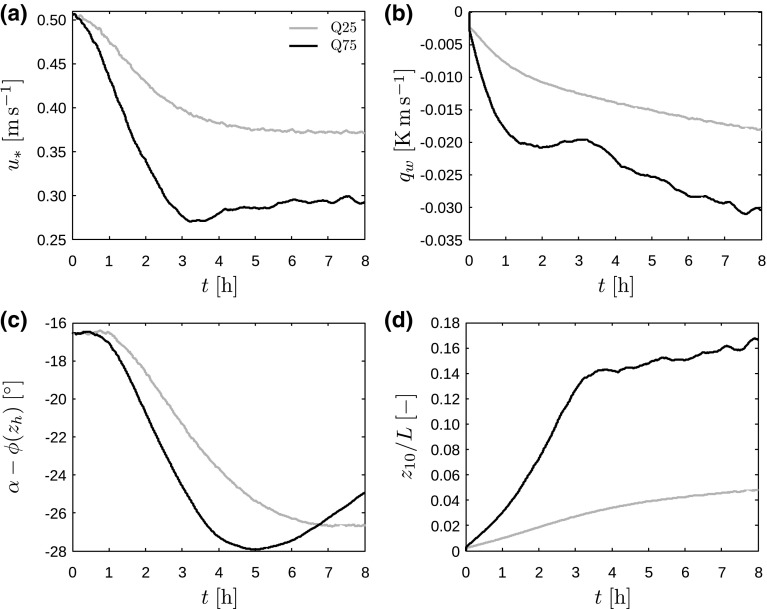
Time evolution of surface-layer characteristics, including **a** friction velocity $u_*$, **b** surface heat flux $q_\mathrm{w}$, **c** difference in wind direction between hub height and free atmosphere $\alpha -\phi (z_\mathrm{h})$, and **d** stability parameter $z_{10}/L$ (using $z_{10}=10\;\mathrm {m}$), for cases Q25 (grey lines) and Q75 (black lines)

### A Growing Nocturnal SBL

The development of a nocturnal boundary layer is simulated by reducing the surface temperature in both the precursor and main domains at a constant rate of 0.25 and $0.75\;\mathrm {K}\,\mathrm {h}^{-1}$ in cases Q25 and Q75, respectively. The smallest cooling rate corresponds to that applied in the GABLS1 benchmark case (Beare et al. 2006), so we expect the transient behaviour to last for about 6–8 h, after which the boundary-layer flow presumably reaches a quasi-steady state. Both simulations are therefore advanced in time for 8 h. Further, a reference case Q00 is included, in which the simulation of a wind farm in conventionally neutral conditions is continued. The boundary layer is in equilibrium in this case, and statistics are collected over a period of 2 h.

The time evolution of some characteristic parameters of the surface layer are shown in Fig. 2 for cases Q25 and Q75. Figure 2a shows that the friction velocity decreases in time as TKE is being destroyed by the stable stratification. The strongest decline thereby corresponds to the highest surface cooling rate. The decrease in friction velocity ceases after about 5 and 3 h in cases Q25 and Q75, respectively, after which it remains roughly constant. In Fig. 2b, the magnitude of the surface heat flux increases in time and is highest for case Q75. It is observed that both the friction velocity and the surface heat flux react immediately to the reduced surface temperature. However, a substantial change in wind direction only occurs after about 30 and 60 min in cases Q75 and Q25, respectively (see Fig. 2c). As expected, the wind near the surface turns towards the pressure gradient and the difference with the geostrophic wind direction increases. In case Q75, the difference in wind direction between hub height and the free atmosphere reaches a maximum of about $28^\circ$ (in absolute value) after 5 h, after which it decreases again. In case Q25, on the other hand, the rate of change only slows down near the end of the simulation. Finally, Fig. 2d displays the stability parameter *z* / *L* (with $L=-\theta _0u_*^3/\kappa g q_\mathrm{w}$ the Obukhov length) evaluated at a height of $z_{10}=10\;\mathrm {m}$. The figure shows that the stability of the surface layer increases over time, and the strongest stability always corresponds to the highest surface cooling rate.

**Fig. 3:**
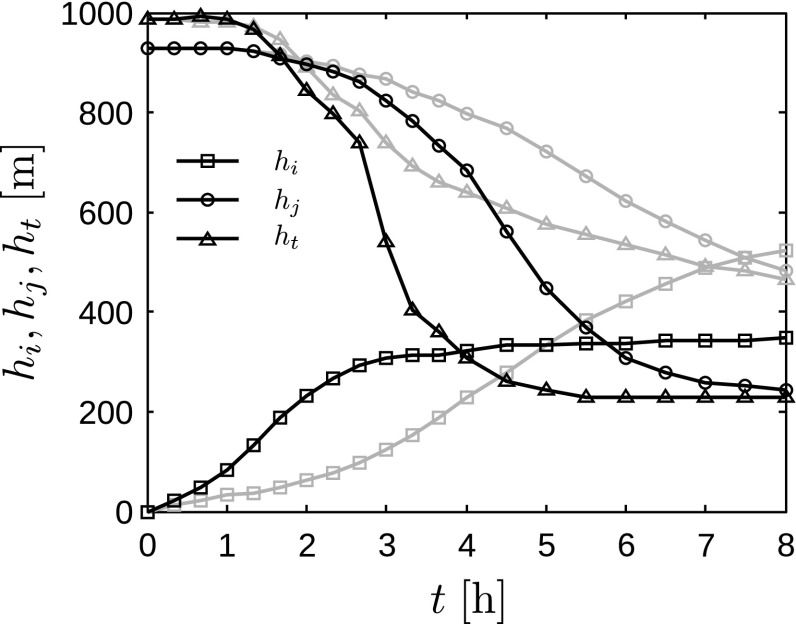
Time evolution of characteristic height scales, including the height of the turbulent layer $h_\mathrm{t}$ (triangles), the height of the surface inversion layer $h_\mathrm{i}$ (squares) and the height of the low-level jet $h_\mathrm{j}$ (circles), for cases Q25 (grey linestyles) and Q75 (black linestyles)

The region above the surface layer can be characterised by three height scales: the height of the turbulent layer $h_\mathrm{t}$, the height of the surface inversion layer $h_\mathrm{i}$ and the height of the low-level jet (LLJ) $h_\mathrm{j}$ (André and Mahrt 1982). First, the height of the turbulent layer represents the level above which all turbulence has decayed. Following Kosović and Curry (2000), an estimate is obtained by linearly extrapolating the height where the turbulent shear stress equals 5% of the wall stress $u_*^2$. Second, the height of the surface inversion layer indicates the height up to which the temperature is affected by the surface cooling. André and Mahrt (1982) define this height as the level where the potential temperature gradient is lower than a given value. Here, the free atmosphere stratification is used as a threshold, i.e., $\partial \theta /\partial z =1\;\mathrm {K}\,\text {km}^{-1}$. Note, however, that the height of the surface inversion is very sensitive to the threshold value applied in its definition. The third measure $h_\mathrm{j}$ characterizes the low-level jet, which is formed by an inertial oscillation of the wind in response to the rapid decay of turbulent fluxes (Blackadar 1957; Shapiro and Fedorovich 2010; van de Wiel et al. 2010). The height of the jet is simply calculated as the vertical level where the horizontal wind speed attains it maximum value.

The temporal evolution of these characteristic heights is depicted in Fig. 3. As the SBL develops in time, the height of the turbulent layer decreases while the height of the surface inversion increases. This was also reported by, e.g., André and Mahrt (1982), Smedman (1991), Kumar et al. (2006). As expected, turbulence decays more rapidly in case Q75 with stronger surface cooling, and the height of the turbulent layer is lower than in case Q25. The surface inversion, on the other hand, rises faster in case Q75 than in case Q25 due to the higher surface heat flux. However, the increase in surface inversion depth levels off in case Q75 after 3 h, while in case Q25 the inversion continues to rise as turbulence levels are higher, and eventually the surface inversion is deeper in case Q25 than in case Q75. We further note that an equilibrium is reached after about 5 h in case Q75 with respect to both $h_\mathrm{t}$ and $h_\mathrm{i}$, whereas the characteristic heights continue to change in case Q25 even after 8 h of cooling.

**Fig. 4:**
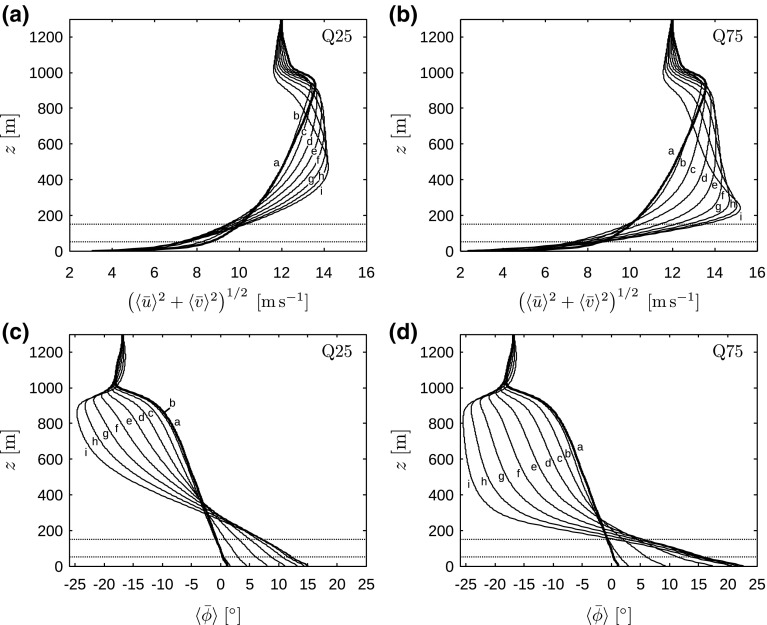
Vertical profiles, averaged over the horizontal directions, of horizontal velocity magnitude (**a**, **b**) and direction (**c**, **d**), for cases Q25 (**a**, **c**) and Q75 (**c**, **d**). Curves **a**–**i** are instantaneous profiles taken every hour between $t=0$ and 8 h. The horizontal dotted lines illustrate the location of the turbine region (note that no wind turbines are simulated in the precursor domain)

The height of the LLJ is shown to decrease over time, which agrees with the finding of Kumar et al. (2006). Moreover, stronger cooling results in a lower LLJ. An explanation is found in the study of Shapiro and Fedorovich (2010), who showed that LLJs have greater wind speeds and occur at lower heights for larger reductions in ambient turbulence levels. Hence, the lower values of $h_\mathrm{j}$ in case Q75 are caused by the stronger reduction of turbulence in this case. We also observe that the LLJ is located just above the height of the turbulent layer near the end of the simulation. This is in accordance with the finding of Shapiro and Fedorovich (2010) that the inertial oscillation amplitude increases towards the ground until frictional forces become important.

The development of the LLJ is further illustrated in Fig. 4, showing vertical profiles of horizontal velocity magnitude and direction for cases Q25 and Q75 at various points in time. Figure 4a, b show that the horizontal velocity decreases near the surface in agreement with the reduced friction velocity observed in Fig. 2a, while above the wind turbine region a low-level jet forms. In case Q75, the LLJ first develops a broad maximum and evolves into a narrow LLJ near the end of the simulation. This finding is in agreement with the study of Kumar et al. (2006), who observed a narrow LLJ only after about 7 h of surface cooling. In case Q25, however, the LLJ remains relatively broad throughout the simulation. We also observe that in case Q25 the LLJ is located relatively high above the turbine region so that the velocities at hub height are lower than the initial values, whereas in case Q75 the LLJ is located closer to the turbine region, which results in significantly higher velocities.

**Fig. 5:**
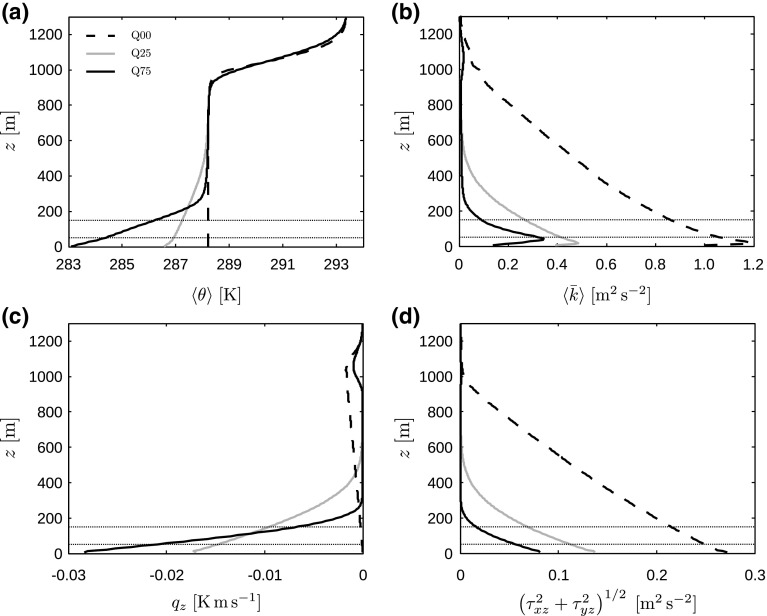
Vertical profiles, averaged over the horizontal directions, of **a** potential temperature $\langle \theta \rangle$, **b** turbulent kinetic energy $\langle \bar{k}\rangle$, **c** total heat flux $q_z$ and **d** total shear stress magnitude $(\tau _{xz}^2+\tau _{yz}^2)^{1/2}$, for cases Q00 (dashed black lines), Q25 (solid grey lines) and Q75 (solid black lines). The potential-temperature profiles are obtained from instantaneous LES data at the end of the simulation, while the second-order statistics (**b**–**d**) have been averaged over the last two simulation hours. The horizontal dotted lines illustrate the location of the turbine region (note that no wind turbines are simulated in the precursor domain)

The changes in velocity magnitude are accompanied by local turning of the wind direction, as shown in Fig. 4c, d. Near the surface, the wind turns towards the pressure gradient (the wind angle increases), whereas in the outer layer the ageostrophic wind speed decreases (the wind angle becomes more negative). This leads to considerable wind veer in the boundary layer, i.e., near the end of the simulation, the total wind veer over the turbine region is $5.2^\circ$ and $13.2^\circ$ in case Q25 and Q75, respectively.

To conclude, Fig. 5 displays the boundary-layer structure at the end of the simulation and compares it with the time-averaged profiles of the CNBL (case Q00). In all figures, it is observed that the largest changes correspond to case Q75 with the strongest surface cooling. Furthermore, a residual layer with constant potential temperature and zero TKE, shear stress and heat flux is clearly visible above the turbulent layer. Even after 8 h of surface cooling, there is still TKE present at the capping inversion, which might be related to shear instability at the top of the LLJ (Taylor and Sarkar 2008).

## Wind-Farm Power and Boundary-Layer Flow

**Fig. 6:**
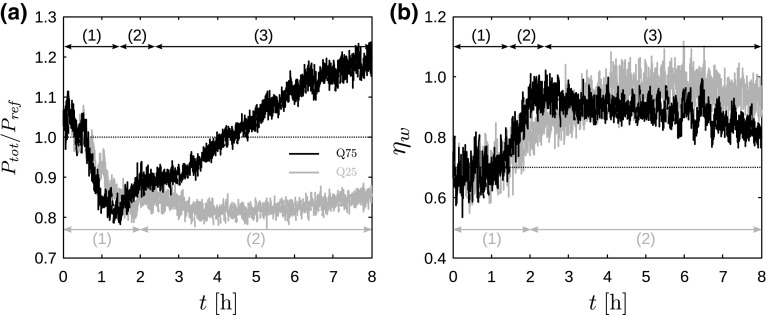
Time evolution of **a** total wind-farm power, normalized by the mean power in conventionally neutral conditions, and **b** wind-farm wake efficiency $\eta _\mathrm{w}$ (see Eq. 2), for cases Q25 (grey lines) and Q75 (black lines). The dotted lines correspond to the wind-farm performance in conventionally neutral conditions. Arrows and numbers in black and grey respectively mark dominant regimes, i.e., (1) a turbulence decay effect; (2) a geometrical effect; and (3) a jet effect

We now look at the results of the main simulation domain. The evolution of the wind-farm power during the boundary-layer transition is shown in Fig. 6 for cases Q25 and Q75. In Fig. 6a, the total wind-farm power is normalised by the mean power in conventionally neutral conditions (i.e., the mean power in case Q00). Figure 6b shows the wind-farm wake efficiency, which is defined as the ratio between the actual power output and the power that would be produced if all turbines were first-row turbines,

2 $$\begin{aligned} \eta _\mathrm{w} = \frac{P_\mathrm{tot}}{N_\mathrm{t}P_1}, \end{aligned}$$

where $N_\mathrm{t}$ is the number of turbines in the farm and $P_1$ is the average power output of a first-row turbine.

In addition to high-frequency oscillations related to turbulence, both wind-farm power output and wake efficiency are slowly varying in time, and a clear steady state is not achieved during the simulated time. Several aspects influence the power performance, and, based on the dominant effects and the differences in time scales, three regimes are identified. The first regime is dominated by a *turbulence decay effect*, leading to reduced hub height velocities and a monotonic decrease in first-row power output. The power of the downstream turbine rows also decreases because less energy is being transported downwards due to the reduced turbulent intensity. Consequently, the total power decreases in the first regime as shown by Fig. 6a. The decrease in wake efficiency caused by the reduced wake recovery is about 5% and is obscured by the large turbulent fluctuations in Fig. 6b. The behaviour of both cases is very similar in this regime, and the power decreases more rapidly in case Q75 due to the stronger reduction in hub-height velocity.

In the second regime, a *geometrical effect* occurs due to the changing wind direction at hub height, i.e., the effective wind-farm layout in the mean flow direction is evolving from an aligned pattern towards a staggered pattern, causing the wind-farm wake efficiency to increase.

In case Q75, the second regime starts after roughly 1.5 h when the hub-height wind direction has turned almost $2^\circ$ with respect to the direction at $t=0$ (see Fig. 2c). For the current wind-turbine spacing ($s_xD=750\;\mathrm {m}$), this angle corresponds to a spanwise wake deflection of *D* / 4 at the next turbine row. The increase in wind-farm wake efficiency more than compensates for the decreasing wind speed, and causes the power to increase in case Q75. In case Q25, on the other hand, regime two only starts after about 2 h, which is in agreement with the slower rotation rate observed in Fig. 2c. The increasing efficiency roughly compensates for the decreasing hub-height velocity, so that the power output remains nearly constant. Finally, we emphasise that the geometrical effect depends on the initial wind-farm layout and does not necessarily always evolve in the direction of increased efficiency. For example, a wind farm whose turbines form a staggered pattern relative to the daytime wind direction would experience a decreasing efficiency as the effective layout changes towards a less efficient, aligned pattern.

In case Q75, a third regime governed by a *jet effect* arises due to the development of a low-level jet with high wind speeds above the wind farm. The hub-height velocity starts to increase after about 2.5 h, causing the power output to rise even further. The wind-farm wake efficiency levels off and slowly decreases in this regime. As mentioned before, the LLJ in case Q25 does not lead to increased wind speeds at hub height, and therefore regime three does not occur for this case.

**Fig. 7:**
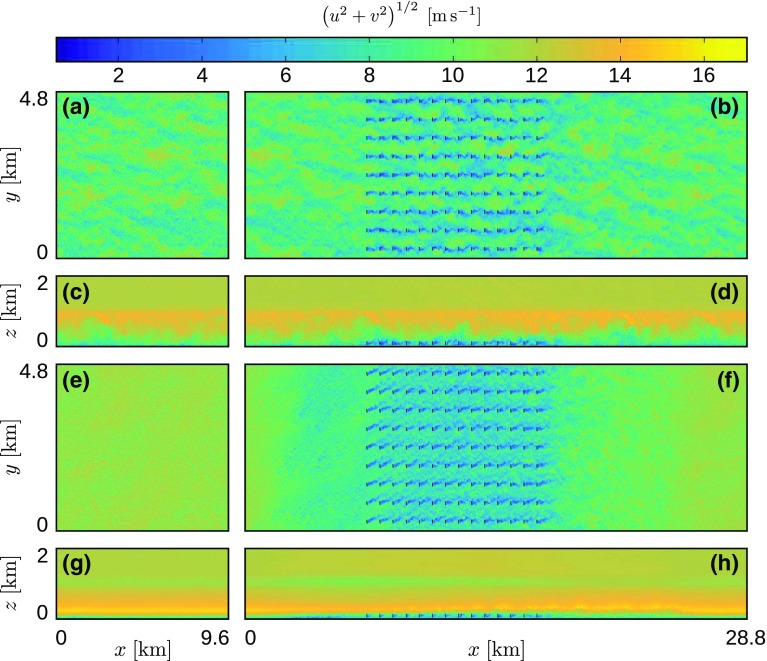
Instantaneous contours of horizontal velocity magnitude at $t=0$ (**a**–**d**), and at $t=8\;\mathrm {h}$ for case Q75 (**e**–**h**); **a**, **b**, **e**, **f** an *x*–*y* plane at turbine hub height $z_\mathrm{h}=100$ m; **c**, **d**, **g**, **h** an *x*–*z* plane through the middle of a turbine column (only the lower 2 km of the numerical domain are shown). The left panel shows the precursor domain (**a**, **c**, **e**, **g**) and the right panel shows the main domain (**b**, **d**, **f**, **h**), where turbine disk locations are indicated with black lines

We now turn our attention to the boundary-layer flow inside the wind farm. A sample of the instantaneous horizontal velocity field in both the precursor and main domain is shown in Fig. 7 for two different times. Figure 7a–d illustrate the statistically steady state of the wind farm in the CNBL at $t=0$. Figure 7a–d show an *x*–*y* plane at hub height and an *x*–*z* plane through the centre of the turbine disks, respectively. The *x*–*y* planes show the existence of elongated velocity streaks, which are typical for neutral boundary layers. In the side view, we observe that the turbulent structures are limited by the overlying inversion layer.

Figure 7e–h show instantaneous contours of horizontal velocity in the same planes but now for case Q75 at $t=8\;\mathrm {h}$. In stable conditions, the turbulent structures are significantly smaller, the elongated velocity streaks have disappeared, and the vertical extent of the turbulent structures in the *x*–*z* planes is severely reduced. In Fig. 7f, we observe a wind-farm wake downstream of the farm where the flow is considerably more turbulent and contains larger turbulent structures. In conventionally neutral conditions, the effect of the farm on the flow downstream is less pronounced (cf. Fig. 7b). Further, Fig. 7f shows that the mean flow decreases significantly upstream of the wind farm, which is related to a strong pressure gradient in that region caused by gravity waves (see Sect. 5). Regarding the wind-turbine wakes, wake meandering appears to be less intense in stable conditions, at least in the first few rows of the farm. España et al. (2011) pointed out that wake meandering is generated by turbulent length scales larger than the wake width, so the reduced meandering could be explained by the absence of large-scale structures in stable conditions.

**Fig. 8:**
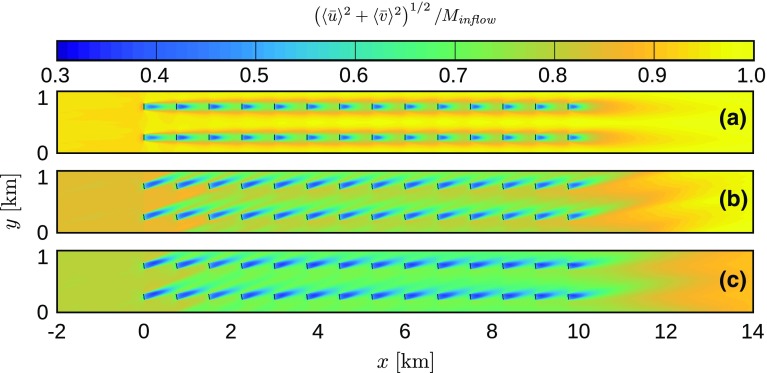
Horizontal velocity magnitude, normalized by the inflow velocity, in an *x*–*y* plane at hub height, averaged in time and per turbine column, for cases **a** Q00 (2-h average), **b** Q25 (30-min average between $t=7.5$ and 8 h) and **c** Q75 (30-min average between $t=7.5$ and 8 h). Results are only shown from 2 km upstream to 4 km downstream of the wind farm

**Fig. 9:**
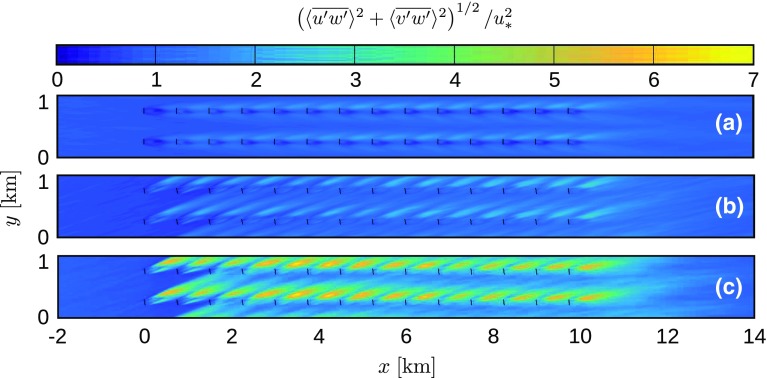
Resolved shear stress, normalized by the square of the friction velocity in the precursor simulation, in an *x*–*y* plane at hub height, averaged in time and per turbine column, for cases **a** Q00 (2-h average), **b** Q25 (30-min average between $t=7.5$ and 8 h) and **c** Q75 (30-min average between $t=7.5$ and 8 h). Results are only shown from 2 km upstream to 4 km downstream of the wind farm

**Fig. 10:**
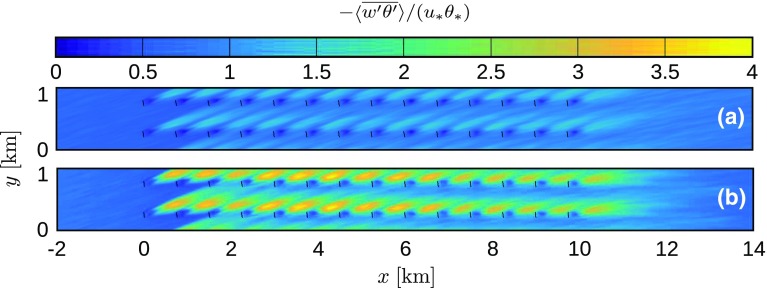
Resolved heat flux, normalized by the surface heat flux in the precursor simulation, in an *x*–*y* plane at hub height, averaged over 30 min between $t=7.5$ and 8 h and per turbine column, for cases **a** Q25 and **b** Q75. Results are only shown from 2 km upstream to 4 km downstream of the wind farm

The mean flow structure is discussed in Figs. 8, 9 and 10. The results of cases Q25 and Q75 are averaged over 30 min between $t=7.5$ and 8 h, while the reference case Q00 is averaged over 2 h. Moreover, since the mean flow structure is periodic in the spanwise direction with a length $s_yD$ (i.e., per turbine column), an average is taken over all the turbine columns as well. The figures depict a horizontal *x*–*y* plane at turbine hub height $z_\mathrm{h}=100\;\mathrm {m}$, in which the mean flow around two turbine columns is shown, allowing a good visualisation of both the wake structure and the channel between the turbine columns.

The horizontal wind speed is shown in Fig. 8, normalized by the inflow velocity. It is clear that the effective farm layout changes in the stable cases as the turbine wakes align with the incoming flow direction. In both cases Q25 and Q75, the first three rows operate in almost unperturbed airflow. However, it is observed both within and downstream of the wind farm that wake recovery is less efficient in stable conditions, causing velocity deficits to extend over longer distances. Further, it is found that the centre line of the turbine wakes (and also the yaw angle of the wind turbines) changes direction throughout the farm, i.e., the wake direction of the last row is rotated about $3.3^\circ$ and $7.1^\circ$ towards the right compared the wake of the first row in cases Q25 and Q75, respectively. This behaviour is explained by the increased turbulence levels in the wind farm (see also Fig. 9), causing enhanced mixing between the wake region and the overlying flow, which has a relative wind direction towards the right (van der Laan and Sørensen 2016). In neutral conditions, the self-generated turbulence by the wind turbines is very small compared to the ambient turbulence intensity (see Fig. 9a), so the wake direction does not change throughout the farm. Note that Allaerts and Meyers (2017) found a change in the opposite direction throughout the wind farm in neutral conditions with lower surface roughness. In their simulations, the directional change is dominated by the velocity reduction and the Coriolis effect inside the farm, and the enhanced mixing with the overlying flow is less important.

Figure 9 depicts the resolved shear stress, normalised by the square of the friction velocity in the precursor simulation. In all simulations, an elevated shear stress level is observed in the wake of the wind turbines. Moreover, the relative increase of the shear stress is highest in the strongest cooling case. Similar trends are observed in the heat flux, which is shown in Fig. 10 (values are normalized by the surface heat flux obtained from the precursor simulation). The heat flux in the neutral case is negligible, so only cases Q25 and Q75 are shown. A strong increase in vertical heat flux is observed in the wake regions, with the largest effects corresponding to case Q75.

## Influence of Atmospheric Gravity Waves

We first look at the excitation of atmospheric gravity waves by the wind farm in Sect. 5.1. Next, the impact of gravity waves on boundary-layer flow and wind-farm performance is investigated in Sect. 5.2, leading to the definition of a non-local wind-farm efficiency.

### Gravity-Wave Excitation

**Fig. 11:**
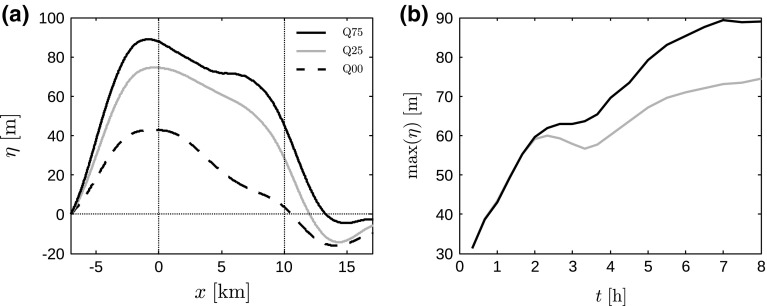
**a** Streamwise variation of inversion-layer displacement $\eta$, averaged over the spanwise direction, for cases Q00 (dashed black line, 2-h average), Q25 (solid grey line, 30-min average between $t=7.5$ and 8 h) and Q75 (solid black line, 30-min average between $t=7.5$ and 8 h); and **b** time evolution of the maximum inversion-layer displacement for cases Q25 (grey line) and Q75 (black line). The vertical dotted lines in **a** indicate the start and end of the wind farm

Atmospheric gravity waves are a well-known wave phenomena in meteorology and are excited by the vertical displacement of stably-stratified flow due to, e.g., topographical effects, frontal passage, etc. Recently, Allaerts and Meyers (2017) showed that flow blockage in large wind farms can lift up the top of the boundary layer, thereby exciting gravity waves in the inversion layer and the free atmosphere. In the current simulations, we observe similar displacement of the inversion layer and associated pressure perturbations. Figure 11a shows the streamwise variation of the inversion-layer displacement $\eta$ for the various cases (obtained at the end of the simulation). The maximum displacement of the inversion-layer height in conventionally neutral conditions (Q00) is found to be 43 m, compared to the 75 m displacement found by Allaerts and Meyers (2017) for lower surface roughness conditions with the same inflow height (cf. their Fig. 15). Moreover, they reported that the maximum displacement occurred near the end of the farm, while the current results indicate a maximum at the farm entrance. In stable conditions, the maximum displacement increases to 75 and 89 m in cases Q25 and Q75, respectively. The induced pressure profiles follow the same trend, i.e., the maximum perturbation is located at the entrance or even upstream of the farm, inducing a favourable pressure gradient inside the farm in all cases (not shown). Comparing these results with linear theory (see further below and in Appendix 2) on a much larger domain shows very good agreement in terms of pressure and boundary-layer displacement levels inside the wind-farm region (not shown). This ensures that we correctly predict the total amount of slow down and pressure build-up in front of the farm and that we find the correct gravity-wave intensity. The only difference is that linear theory predicts smaller gradients but over larger upstream distances, while in the LES output the slow down is compressed in a smaller upstream region. It may be interesting to explore alternative ways to impose boundary conditions that eliminate this upstream discrepancy without the need for larger computational domains (which is currently computationally not feasible). This is an interesting topic for future research.

The evolution of the flow blockage and the gravity-wave excitation in time, quantified by the maximum inversion-layer displacement, is illustrated in Fig. 11b for cases Q25 and Q75. We observe an almost monotonic increase in gravity-wave excitation with time in both cases. Moreover, the maximum displacement is equal in the beginning for both cases, but starts to diverge after 2 h of surface cooling. In the traditional view of wind-farm–ABL interactions, without feedback of gravity waves through induced pressure gradients, it is expected that the highest displacement corresponds to the case with the largest flow blockage, i.e., the largest relative reduction in wind speed caused by the total thrust force of the wind turbines and the surface. However, neither the total wind-farm power nor the wind-farm wake efficiency match the temporal behaviour of the maximum inversion-layer displacement (compare Figs. 6 and 11). Hence, the time evolution and the observed differences between case Q25 and Q75 are not explained by the relative flow blockage of the wind farm only (without effects of gravity-wave feedback).

Insight into the wind farm–gravity wave system can be gained by analysing a linear one-dimensional model for the boundary-layer displacement $\eta$ in response to an applied drag force (see Appendix 2 for a derivation),

3 $$\begin{aligned} \left( 1-\textit{Fr}^{-2}\right) \frac{\partial \eta }{\partial x}= c_\mathrm{t}{\Pi }(x)-2 \left( c_\mathrm{t}{\Pi }(x)+c_\mathrm{d}\right) \frac{\eta }{H}-P_N^{-1}\mathscr {G}(\eta ), \end{aligned}$$

with ${\Pi }(x)$ a unit pulse equal to one inside the wind farm and zero everywhere else. We identify four physical aspects in this model. First, the left-hand side of Eq. 3 corresponds to the balance between flow acceleration or deceleration and the pressure gradient imposed by the displacement of the inversion layer. This balance is characterised by the Froude number $\textit{Fr}=\overline{U}/\sqrt{g^\prime H}$, with *H* the height of the boundary layer, $\overline{U}$ the boundary-layer bulk velocity in the *x*-direction and $g^\prime =g\varDelta \theta /\theta _0$ a reduced gravity accounting for the inversion strength. A Froude number close to unity means that any change in bulk velocity is almost exactly balanced by a pressure gradient arising from the flow divergence. The pressure induced by vertically-propagating gravity waves forms a second aspect. The magnitude of this effect is expressed by the dimensionless number $P_N=\overline{U}^2/(NU_gH)$, and $\mathscr {G}(\eta )$ is a linear operator that tends to disperse the displacement in space (see Appendix 2 for a definition). The last two elements in the 1D model are the drag by the surface and the wind farm, represented by the drag coefficients $c_\mathrm{d}$ and $c_\mathrm{t}$, respectively.

**Fig. 12:**
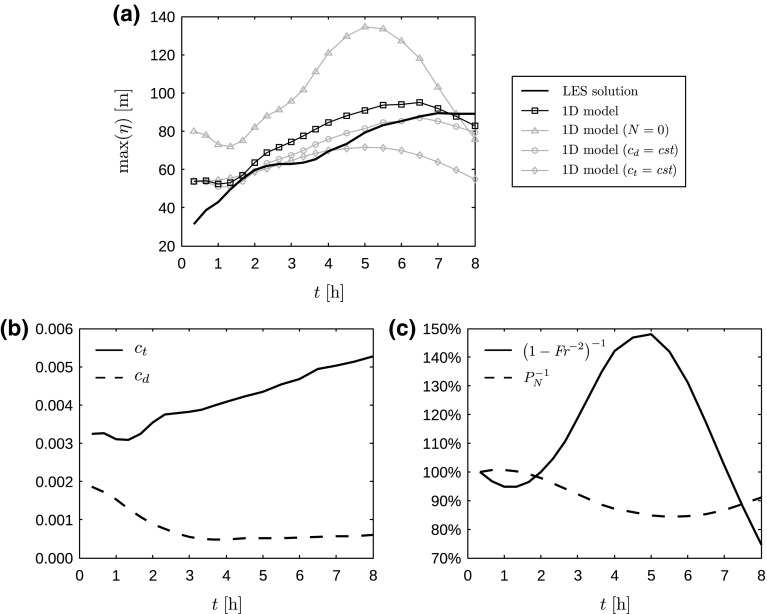
**a** Maximum inversion displacement as a function of time for case Q75, computed with LES and various versions of the simplified one-dimensional model (Eq. 3); and temporal evolution of **b** the drag coefficients $c_\mathrm{d}$ and $c_\mathrm{t}$ and **c** the terms $(1-\textit{Fr}^{-2})^{-1}$ and $P_N^{-1}$ (in percentage) appearing in the model

We focus on case Q75 and compare the maximum displacement obtained with LES to the predictions of the one-dimensional model. Results are shown in Fig. 12a. The parameters $\textit{Fr}$, $P_N$ and $c_\mathrm{d}=u_*^2/\overline{U}^2$ are thereby calculated from the undisturbed precursor simulation, and the wind-farm drag coefficient is computed as (see Appendix 2)

4 $$\begin{aligned} c_\mathrm{t} = \frac{1}{2}\frac{\pi C_\mathrm{T}}{4s_xs_y}\,\eta _\mathrm{w}\,\gamma . \end{aligned}$$

Here, turbine wake effects and wind profile convexity are taken into account via the wake efficiency $\eta _\mathrm{w}$ and the shape factor $\gamma =u_r^2/\overline{U}^2$, with $u_r$ the rotor-averaged wind speed in the precursor simulation. The time evolution of the drag coefficients and the terms $(1-\textit{Fr}^{-2})^{-1}$ and $P_N^{-1}$ are shown in Fig. 12b, c. The linear 1D model is able to predict the trend in the maximum inversion displacement relatively well with a mean absolute error of 9.5 m. Further, we find that the fluctuations in the Froude number are mainly caused by an increase in the bulk velocity $\overline{U}$ due to the inertial oscillation above the SBL (see Sect. 3). The increase in $c_\mathrm{t}$ is related to the increasing wake efficiency $\eta _\mathrm{w}$ (due to the geometrical effect) and the deformation of the velocity profile by the LLJ (see Fig. 4b), causing a strong increase in the shape factor $\gamma$.

We now use the model to determine what aspects dominate the increase in maximum inversion displacement. Smith (2010) investigated wind-farm gravity waves with a very similar model and hypothesised that low surface drag in combination with a Froude number approaching unity could lead to very large displacements (an effect called *choking*). The Froude number in our simulations is indeed close to unity (between 0.83 and 0.9) and the surface drag coefficient is decreasing in time due to turbulence decay (see Fig. 12b). In order to investigate this hypothesis, we use the 1D model keeping the drag coefficient constant to the initial neutral value. Figure 12a shows that the result does not vary significantly when $c_\mathrm{d}$ is kept constant, indicating that the reduction in surface drag does not explain the observed trend.

Next, we investigate the effect of the vertically-propagating gravity waves by excluding them from the model, i.e., by setting $N=0$. Figure 12a shows that this model set-up yields much larger displacements, and the temporal evolution is highly correlated with the Froude number (see, e.g., the significant decrease in the last 3 h in Fig. 12a, c). This result indicates that the vertically-propagating waves play a dominant role by dispersing the choking effect of inversion waves related to a Froude number close to one. We also checked the model output when $P_N$ is kept constant to the initial neutral value, but this has a negligible effect (not shown).

Finally, we investigate the importance of the increase in $c_\mathrm{t}$. Keeping $c_\mathrm{t}$ constant to its initial value, the 1D model strongly underpredicts the inversion displacement, especially in the last 3 h (see Fig. 12a). This shows that the changing wind-farm wake efficiency and the changing shape factor have an important effect on the overall gravity-wave excitation.

We conclude that the choking effect discussed by Smith (2010), related to the Froude number approaching unity, is significantly weakened as a result of the Brunt–Väisälä frequency *N* being nonzero, i.e., related to the presence of vertically-propagating gravity waves. At the same time, the rising wind-farm drag plays a dominant role in the increased gravity-wave excitation. The decreasing surface drag is thereby of minor importance. Finally, we remark that we found this analysis also to hold for case Q25 (results not shown here), i.e., the observed increase is caused by an interplay between wind-farm drag, Froude number and dispersion by free gravity waves, while surface drag has only a minor influence.

### Non-local Wind-Farm Efficiency

**Fig. 13:**
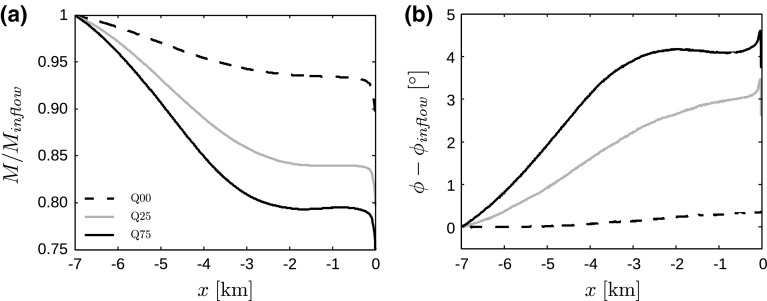
Upstream influence of gravity waves on **a** hub height wind speed, normalized by the inflow velocity, and **b** wind direction, for cases Q00 (dashed black line, 2-h average), Q25 (solid gray line, 30-min average between $t=7.5$ and 8 h) and Q75 (solid black line, 30-min average between $t=7.5$ and 8 h). The results have been averaged over the spanwise direction

The pressure gradients induced by the gravity waves have a profound impact on the upstream flow characteristics as illustrated in Fig. 13, showing the wind speed, normalized by the inflow velocity, and the change in wind direction at hub height (averaged over the spanwise direction and over the last 30 min). It is found that the unfavourable pressure gradients in front of the farm lead to a strong flow deceleration, which then causes the wind to turn towards the left (i.e., towards the large-scale pressure gradient vector and away from the geostrophic wind vector). In line with Fig. 11a, the upstream influence of gravity waves increases with increasing surface-layer stability. With respect to the inflow velocity, a reduction of 6.6, 16 and 20.5% is observed in cases Q00, Q25 and Q75, respectively. Moreover, the decrease in velocity leads to wind direction changes of $3^\circ$ and $4^\circ$ in cases Q25 and Q75, respectively. We reiterate that the level of upstream slowdown is correct, but the slowdown is compressed in a smaller region in the LES output as compared to linear theory (cf. discussion in Sects. 2 and 5.1).

The flow deceleration in front of the wind farm has strong implications on the wind-farm power production, i.e., the power does not only depend on the wake recovery inside the farm but also on the non-local boundary-layer deceleration. In order to quantify the latter effect, we define the non-local wind-farm efficiency as the ratio between the energy extraction in a first row turbine and the theoretical energy extraction by an identical turbine in isolated conditions,

5a $$\begin{aligned}&\displaystyle \eta _\mathrm{nl} = \frac{P_1}{P_\mathrm{th}},\end{aligned}$$

5b $$\begin{aligned}&\displaystyle P_\mathrm{th}= \frac{1}{2}C_\mathrm{p} U_\infty ^3 \frac{\pi }{4}D^2, \end{aligned}$$

with $U_\infty$ the undisturbed velocity averaged over the rotor area obtained from the precursor simulation, and $C_\mathrm{p}$ the power coefficient. Here, we simply relate $C_\mathrm{p}$ to the disk-based thrust coefficient $C_\mathrm{T}^\prime$ using idealized momentum theory (see,e.g., Bokharaie et al. 2016), yielding

6 $$\begin{aligned} C_\mathrm{p} = \frac{64C_\mathrm{T}^\prime }{\left( C_\mathrm{T}^\prime +4\right) ^3}. \end{aligned}$$

Combining the non-local efficiency with the wind-farm wake efficiency introduced before (see Eq. 2), we find that the average energy extraction by a wind turbine located in a large farm is given by

7 $$\begin{aligned} P_\mathrm{avg} \equiv \frac{P_\mathrm{tot}}{N_\mathrm{t}} = \eta _\mathrm{w} \eta _\mathrm{nl} P_\mathrm{th}. \end{aligned}$$

**Fig. 14:**
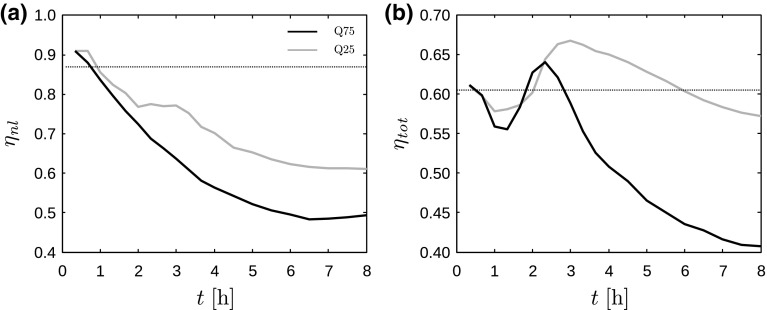
**a** Non-local efficiency $\eta _\mathrm{nl}$ (see Eq. 5a) and **b** total efficiency $\eta _\mathrm{tot}=\eta _\mathrm{w} \eta _\mathrm{nl}$ as a function of time (averaged over consecutive time windows of 20 min), for cases Q25 (gray lines) and Q75 (black lines). The dotted lines correspond to the wind-farm efficiency in conventionally neutral conditions

Figure 14 shows the evolution of the non-local and total wind-farm efficiency as a function of time for the various cases. In line with the results of Figs. 11 and 13, the non-local efficiency decreases with increasing surface-layer stability and time due to the increased excitation of gravity waves and the associated influence on upstream flow conditions. Figure 14b shows that, in neutral conditions, a wind turbine located inside the farm extracts on average almost 40% less energy than an isolated turbine. In stable conditions, the total power deficiency can be as high as 59%, even though the wake loss is only 18%. Other wind-farm layouts may yield considerably higher wake losses in stable conditions, leading to even lower wind-farm efficiencies. However, we remark that the current results obtained with an “infinitely” wide wind farm should be interpreted with care. In a real wind farm of finite width, the flow blockage and gravity-wave excitation will be lower as the wind can flow around the farm, resulting in a higher non-local efficiency. The effect of gravity waves in fully finite wind farms remains subject to further research.

## Energy Flux Analysis

To further understand the wind-farm efficiency and the role of gravity waves, we investigate the energy fluxes through the boundary layer. To this end, we consider the budget equation for the resolved kinetic energy (per unit mass) averaged over the last two simulation hours. Taking the inner product of the filtered Navier–Stokes equations (i.e., Eq. 11 in Appendix 1) and the filtered velocity vector and subsequently averaging in time yields (omitting the LES filtering “tilde” to simplify notation),

8 $$\begin{aligned} \frac{\partial \overline{F}_{E,j}}{\partial x_j} = \frac{g}{\theta _0}\overline{u_3(\theta -\theta _0)} + \overline{\tau ^r_{ij}S_{ij}}+\overline{u_if_i}-\frac{\bar{u}_i}{\rho _0}\frac{\partial p_\infty }{\partial x_i}, \end{aligned}$$

where

9 $$\begin{aligned} \overline{F}_{E,j}=\bar{u}_j\overline{E}_\mathrm{k} + \bar{u}_j\bar{p}^\star /\rho _0+\bar{u}_i\overline{u_i^\prime u_j^\prime }+\frac{1}{2}\overline{u_i^\prime u_i^\prime u_j^\prime }+\overline{u_j^\prime p^\prime }/\rho _0+\overline{u_i\tau ^r_{ij}} \end{aligned}$$

is the total energy flux vector field and the primes denote resolved turbulent fluctuations (w.r.t. the time-averaged value). The first term on the right-hand side of Eq. 9 represents the mean-flow transport of kinetic energy, with $\overline{E}_\mathrm{k}=(\bar{u}_i\bar{u}_i + \overline{ u_i^\prime u_i^\prime })/2$ the sum of the mean-flow and resolved TKE. The second term in Eq. 9 involves the product of the time-averaged pressure and the reciprocal of the density, which is also encountered in the classic thermodynamic definition of enthalpy (i.e., $h=e+p/\rho$ with *h* the enthalpy and *e* the internal energy). We therefore define the second term of Eq. 9 as the mean-flow transport of enthalpy (note that the Boussinesq approximation implies a constant density $\rho _0$). The next three terms in Eq. 9 represent the transport of energy by resolved turbulent fluxes and pressure fluctuations, and the last term corresponds to energy transport by means of subgrid-scale fluxes.

**Fig. 15:**
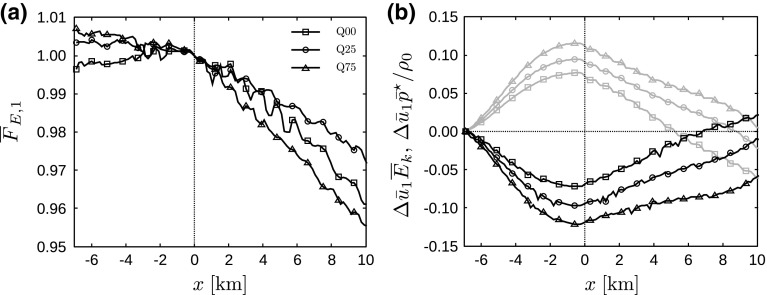
**a** Total energy flux in the *x*-direction $\overline{F}_{E,1}$ (see Eq. 9), integrated from the ground up to the capping inversion and **b** contributions of kinetic energy $\bar{u}_1\overline{E}_\mathrm{k}$ (black linestyles) and enthalpy $\bar{u}_1\bar{p}^\star /\rho _0$ (grey linestyles), averaged over the last 2 h and per turbine column, for cases Q00 (squares), Q25 (circles) and Q75 (triangles). All results are normalized by the total energy flux at the entrance of the farm ($x=0\;\mathrm {km}$), and in **b** the relative change with respect to the inflow ($x=-7\;\mathrm {km}$) is shown

Allaerts and Meyers (2017) found that gravity waves affect the kinetic energy budget by inducing pressure perturbations, which lead to energy conversion between kinetic energy and enthalpy (i.e., energy in the form of pressure). This process is demonstrated in Fig. 15, showing the total energy flux in the *x*-direction integrated from the ground up to the capping inversion, and the contributions of kinetic energy and enthalpy (excluding the mean background pressure). The other contributions to the energy flux are negligible (i.e., less than 3% of the total energy flux) and therefore not shown. Similar to the results of Allaerts and Meyers (2017) (cf. their Fig. 20), the total energy flux is roughly constant upstream of the farm and decreases almost linearly inside the farm. In the current simulations, only small reductions in energy flux are observed, i.e., 3.9, 2.6 and 4.3% in case Q00, Q25 and Q75, respectively. Further, Fig. 15b shows that up to 12% of the energy flux can be present in the form of enthalpy. Conforming to the results discussed earlier, we find that more kinetic energy is being converted into enthalpy with increasing surface-layer stability. Finally, we observe that all energy stored in the pressure field is being released again before reaching the end of the wind farm. Moreover, the total kinetic energy flux is increasing throughout the farm, which indicates that the total boundary-layer flow beneath the capping inversion is accelerating in the wind-farm region. This is in accordance with the observed decrease in inversion-layer height above the wind farm (see Fig. 11a) and is related to the fact that the total mass flux must remain constant.

**Fig. 16:**
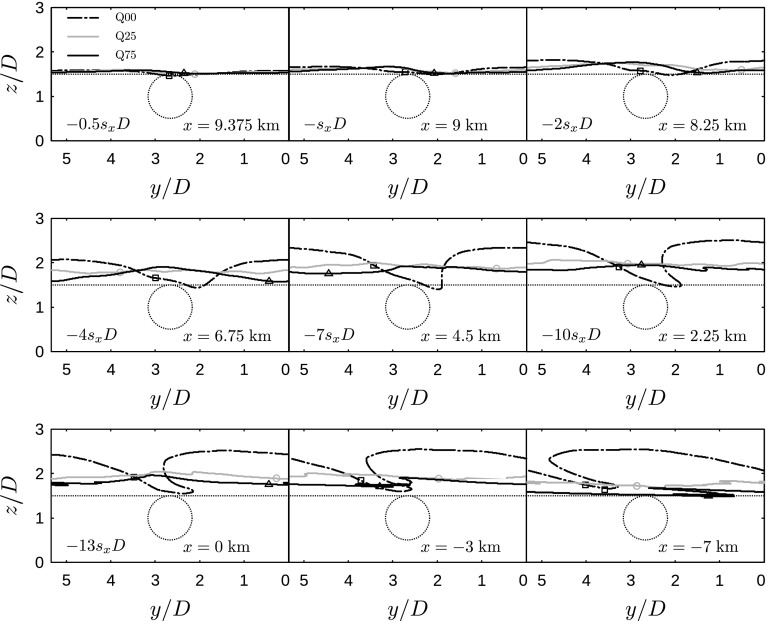
Intersection of *y*–*z* planes at various *x* locations with the surface bounding the energy attraction region (computed from LES data averaged over the last 2 h and per turbine column), for cases Q00 (dashed black lines, squares), Q25 (solid grey lines, circles) and Q75 (solid black lines, triangles). Only one turbine column is shown. Both the relative distance from the last turbine row (left) as well as the *x* location relative to the farm entrance (right) are indicated. The dotted lines indicate the rotor disk area and the initial line above the last turbine row, and the symbols correspond to the energy-transport line that passes through the top of the rotor disk area in the various cases

The energy fluxes in Fig. 15 give an impression of the overall energy household in the region below the capping inversion. In conventionally neutral conditions, this region is entirely filled with turbulence and coincides with the neutral boundary layer. In the stably-stratified cases, however, the region below the capping inversion consists of the stable turbulent boundary layer and the residual layer. As the residual layer is non-turbulent and therefore decoupled from the wind-farm region, Fig. 15 fails to indicate how much of the enthalpy is actually available for the wind farm. To further investigate the energetic consequences of gravity waves, we use the energy tube analysis discussed by Meyers and Meneveau (2013). This method is a generalization of the classical stream tube analysis and constructs energy-transport lines based on the total energy flux vector field (i.e., Eq. 9). These lines enable the visualization of the path taken by the energy flux to arrive at a certain location.

Using the energy tube approach, we restrict our energy budget analysis to the region from which energy is effectively being extracted by the wind turbines. To this end, we construct a surface that passes through a horizontal line above the last turbine row ($x=9.75\;\mathrm {km}$) at a height $z_\mathrm{h}+D/2$. By definition, no energy is being transported through this surface, so all energy extracted by the wind farm must come from within the region bounded by the surface and the ground, which we call the *energy attraction region*. Figure 16 shows intersects of the surface with *y*–*z* planes at various *x* locations for the different cases. In case Q25 and Q75, the surface has been computed from data averaged over the last two simulation hours. First of all, Fig. 16 shows that the wind farm only extracts energy from a very shallow part of the boundary layer, i.e., at the farm entrance ($x=0\;\mathrm {km}$), the maximum height of the energy attraction region is only 252, 204 and 196 m in cases Q00, Q25 and Q75, respectively. In the conventionally neutral case, the energy attraction region attains an asymmetric, lobed structure starting at approximately 4–7 turbine rows upstream of the last row. In front of the farm, the lobe further expands in the positive *y*-direction and the maximum height remains roughly constant. The shape of the energy attraction region is related to the turbulent transport of energy from higher up where the wind speed is higher and directed towards the right (in relative terms).

In the stable cases, on the other hand, the top of the energy attraction region is lower and contains much smaller spanwise perturbations due to the reduced turbulence levels. In contrast to the neutral case, the maximum height decreases in front of the farm to 184 and 168 m in case Q25 and Q75, respectively. This behaviour is caused by the significant flow blockage upstream of the farm (see also Fig. 13a) which deflects the flow upwards, i.e., the downward energy transport by means of turbulence above the wind farm is only compensating for the upward transport of energy in front of the farm and transports almost no energy from regions higher up towards the farm.

**Fig. 17:**
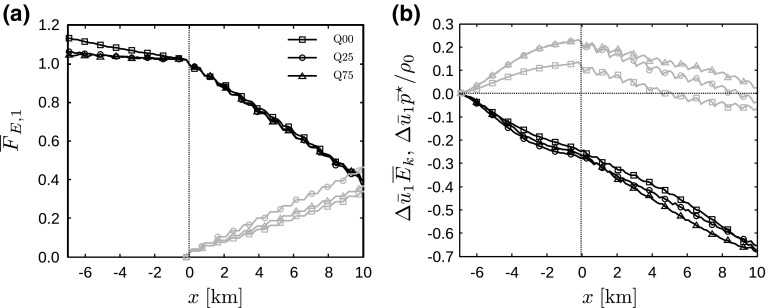
**a** Total energy flux in the *x*-direction $\overline{F}_{E,1}$ (see Eq. 9), integrated over the energy attraction region (black linestyles) and cumulative wind-farm energy extraction (grey linestyles), and **b** contributions of kinetic energy $\bar{u}_1\overline{E}_\mathrm{k}$ (black linestyles) and enthalpy $\bar{u}_1\bar{p}^\star /\rho _0$ (grey linestyles), averaged over the last 2 h and per turbine column, for cases Q00 (squares), Q25 (circles) and Q75 (triangles). All results are normalized by the total energy flux at the entrance of the farm ($x=0\;\mathrm {km}$), and in **b** the relative change with respect to the inflow ($x=-7\;\mathrm {km}$) is shown

We can now integrate the energy fluxes from the ground up to the top of the energy attraction region, and the results are presented in Fig. 17. We observe a small decrease in the energy flux level upstream followed by a strong decrease inside the wind farm in Fig. 17a. Interestingly, we find that the reduction in energy flux through the energy attraction region is equal for all cases, with a total reduction of almost 60% over the entire wind farm. Figure 17a also shows that the wind farm accounts for 34, 46 and 36% of the decrease in energy flux in case Q00, Q25 and Q75, respectively. The remaining energy release is balanced by dissipation, conversion into potential energy and work by the mean background pressure (see Eq. 8).

Figure 17b shows the dominant components of the energy flux, i.e., the transport of mean kinetic energy and enthalpy. The enthalpy flux increases by 23% from the inlet to the farm entrance in the stable cases, after which it releases its energy throughout the farm. In the neutral case, the total rise in enthalpy flux is only 14%. On the other hand, the kinetic energy flux is reduced by 25–28% upstream of the farm and decreases even more inside the farm. This indicates that the previously observed kinetic energy increase in the wind-farm sector in Fig. 15b is restricted to the layer between the attraction region and the inversion layer.

Finally, we remark that the observed rise and drop in enthalpy flux implies a coupling between the non-local efficiency and wake efficiency introduced before. The upstream flow deceleration responsible for the non-local efficiency is converted into enthalpy flux, which is released again throughout the farm. This can affect turbine wake recovery and wind-farm wake efficiency. Thus, a low non-local efficiency may lead to a higher wake efficiency.

## Conclusion

The aim of the present study was to examine the impact of stable stratification on the performance of large wind farms and the role of wind-farm induced gravity waves. To this end, we performed large-eddy simulations of a large wind farm during the transition from conventionally neutral to stable conditions. Two different cooling rates were applied for a period of 8 h and compared with a neutral reference case.

Streamwise flow development through the wind farm and non-local effects both upstream and downstream of the farm were studied by using a very large numerical domain in combination with the concurrent-precursor method. Inflow conditions were thereby generated in a separate precursor domain, which simulated the flow transition due to an applied cooling rate in the absence of wind turbines. Analysis of the stable boundary-layer development in the precursor simulation showed good comparison with the literature, and revealed three important processes. The process with the smallest time scale was the decay of turbulence, related to the destruction of turbulent kinetic energy by buoyancy. The sudden drop in turbulent momentum flux reduced the wind speed close to the surface and initiated an inertial oscillation, which in turn caused two additional processes. The oscillation first leads to significant changes in wind direction, followed by the development of a narrow low-level jet with high wind speeds above the wind farm.

These three processes affecting the wind speed and wind direction at hub height were reflected in the wind-farm performance. The turbulence decay effect first resulted in reduced output of first-row turbines and less efficient wake recovery. Subsequently, a geometrical effect occurred due to the changing wind direction and the accompanying modification of the effective wind-farm lay-out. The particular choice of an initially aligned wind-turbine pattern thereby resulted in an increase of the wind-farm wake efficiency. Finally, the jet effect started to accelerate the velocity at hub height in the moderately stable case after 2.5 h, and increased the power output in this case significantly.

The wind farm was found to excite atmospheric gravity waves in all cases, and the amplitude of the waves increased with time and surface-layer stability. Analysis of a simplified one-dimensional model showed that the decrease in surface drag due to turbulence decay does not explain the observed increase in gravity-wave excitation, contrary to what was postulated before by Smith (2010). Instead, the gravity-wave intensity was related to an interplay between the rising wind-farm drag, the fluctuations in the Froude number and the dispersive properties of the vertically-propagating gravity waves. Moreover, the variations in wind-farm drag and Froude number were directly related to the changes in the boundary-layer bulk velocity and the wind profile convexity, indicating that inertial oscillations have a profound effect on the intensity with which wind farms excite atmospheric gravity waves.

An important consequence of wind-farm gravity waves is that the induced pressure gradients lead to significant flow deceleration upstream of the farm. As a result, the average power output of the first-row turbines was lower than the theoretical output of an isolated turbine, which was quantified by introducing the non-local wind-farm efficiency. We found that the total wind-farm efficiency can be as low 41%, even though the wake loss only accounts for 18%. Further analysis of the energy fluxes through the boundary layer showed that wind farms only extract energy from a very shallow part of the boundary layer (only from the lowest 252 m in our neutral case and even lower in stable conditions). Furthermore, we showed that the turbine wake recovery and the non-local efficiency are coupled.

The current study was limited to a single wind-farm layout due to computational constraints. However, the observed trends in the wake efficiency (e.g., related to the geometrical effect) and hence in the excitation of gravity waves are highly dependent on the chosen layout. The quantitative results for varying surface-layer stability can therefore not be extrapolated to other wind-farm layouts, and more research is required to determine the sensitivity to the wind-farm layout. Further, we considered the asymptotic limit of an “infinitely” wide wind farm. In this configuration, the streamwise flow deceleration inside the wind farm is entirely converted into upward flow deflection, leading to significant boundary-layer displacement and gravity-wave excitation. In real wind farms, the wind can also flow around the farm and non-local effects will have a lower magnitude and a reduced horizontal extent. Given the detrimental effect of upstream flow deceleration on the wind-farm efficiency, further research into gravity waves excited by fully finite wind farms is needed. Next to this, applying Rayleigh damping at the top of the domain to limit gravity-wave reflection required very large vertical domain sizes. Therefore, research into more advanced methods that limit gravity-wave reflections at the domain boundaries is strongly recommended. Finally, we note that the free atmosphere lapse rate has a strong impact on the intensity of atmospheric gravity waves, and it would be interesting to assess the sensitivity of wind-farm performance to this parameter.

## Appendix 1: LES Methodology

This section briefly outlines the large-eddy simulation methodology used to study wind farms in stable atmospheric conditions.

**Governing flow equations** The three-dimensional flow field is found by solving the filtered transport equations for velocity and potential-temperature, $\tilde{u}_i$ and $\tilde{\theta }$, and the continuity equation for incompressible flow, i.e.,

10 $$\begin{aligned}&\frac{\partial \tilde{u}_i}{\partial x_i}=0, \end{aligned}$$

11 $$\begin{aligned}&\frac{\partial \tilde{u}_i}{\partial t} + \tilde{u}_j \frac{\partial \tilde{u}_i}{\partial x_j} = \delta _{i3}g\frac{\tilde{\theta }-\theta _0}{\theta _0} + f_\mathrm{c}\epsilon _{ij3}\tilde{u}_j -\frac{\partial \tau ^r_{ij}}{\partial x_j} -\frac{1}{\rho _0}\frac{\partial \tilde{p}^\star }{\partial x_i} -\frac{1}{\rho _0}\frac{\partial p_\infty }{\partial x_i} -f_i, \end{aligned}$$

12 $$\begin{aligned}&\frac{\partial \tilde{\theta }}{\partial t}+\tilde{u}_j\frac{\partial \tilde{\theta }}{\partial x_j}=-\frac{\partial q^{R}_j}{\partial x_j}, \end{aligned}$$

where index $i=1,2$ and $i=3$ correspond to the horizontal and vertical coordinate directions, respectively. The first term on the right-hand side of Eq. 11 is the buoyancy force (using the Boussinesq approximation), with *g* the acceleration due to gravity, and $\theta _0$ and $\rho _0$ the temperature and density of the adiabatic base state. The second term represents the Coriolis force, with $f_\mathrm{c}=2\varOmega \sin \phi$ being the Coriolis parameter, $\varOmega$ the angular velocity of the earth and $\phi$ the latitude. Further, $q_j^R$ is the subgrid-scale heat flux and $\tau _{ij}^R$ represents the subgrid-scale stress tensor, of which the anisotropic part $\tau _{ij}^r=\tau _{ij}^{R}-\delta _{ij}\tau _{kk}^R/3$ is modelled (see further below). The trace of the subgrid-scale stress tensor is included in the filtered modified pressure, i.e., $\tilde{p}^\star =\tilde{p}-p_\infty +\rho _0\tau _{kk}^R/3$, with $p_\infty$ a mean background pressure. Finally, $f_i$ represents the drag force exerted by the wind turbines on the boundary layer (see further below).

The flow is driven by applying a mean pressure gradient $\nabla p_\infty$, which relates to the geostrophic wind speed *G* by the geostrophic balance for barotropic conditions,

13a $$\begin{aligned}&\displaystyle \frac{1}{\rho _0}\frac{\partial p_\infty }{\partial x}=f_\mathrm{c}G\sin \alpha ,\end{aligned}$$

13b $$\begin{aligned}&\displaystyle \frac{1}{\rho _0}\frac{\partial p_\infty }{\partial y}=-f_\mathrm{c}G\cos \alpha , \end{aligned}$$

with $\alpha$ the angle between the geostrophic velocity vector and the *x*-direction. In the current study, the LES filtering “tilde” is often omitted to simplify notation.

**Subgrid-scale model** The anisotropic part of the subgrid-scale stress tensor is obtained from the resolved velocity field with an eddy-viscosity model, i.e.,

14 $$\begin{aligned} \tau _{ij}^{r} = -2K_{\textit{sgs}}S_{ij}, \end{aligned}$$

with $S_{ij} = 0.5\left( \partial \tilde{u}_i/\partial x_j+\partial \tilde{u}_j/\partial x_i\right)$ the filtered rate of strain and $K_{\textit{sgs}}$ the subgrid-scale eddy viscosity. The subgrid-scale heat flux is calculated in a similar manner from the resolved potential temperature profile using an eddy-diffusivity model

15 $$\begin{aligned} q_{j}^{r} = -K_{\textit{sgs}}\textit{Pr}_{\textit{sgs}}^{-1}\frac{\partial \tilde{\theta }}{\partial x_j}, \end{aligned}$$

with $\textit{Pr}_{\textit{sgs}}$ the subgrid-scale Prandtl number. The eddy coefficients are computed with the turbulent kinetic energy model developed by Deardorff (1980), which defines the eddy viscosity as

16 $$\begin{aligned} K_{\textit{sgs}}= c_ml\sqrt{k_{\textit{sgs}}}. \end{aligned}$$

The subgrid-scale turbulent kinetic energy $k_{\textit{sgs}}\equiv \frac{1}{2}\tau _{ii}^R$ follows from the additional transport equation

17 $$\begin{aligned} \frac{\partial k_{\textit{sgs}}}{\partial t}+\tilde{u}_j\frac{\partial k_{\textit{sgs}}}{\partial x_j}=K_{\textit{sgs}}S^2-K_{\textit{sgs}}\textit{Pr}_{\textit{sgs}}^{-1}N^2+\frac{\partial }{\partial x_j}\left( 2K_{\textit{sgs}}\frac{\partial k_{\textit{sgs}}}{\partial x_j}\right) -c_\epsilon \frac{k_{\textit{sgs}}^{3/2}}{l}, \end{aligned}$$

where $S=\left( 2S_{ij}S_{ij}\right) ^{1/2}$ is the characteristic filtered rate of strain and $N=[(g/\theta _0) \,\partial \tilde{\theta }/\partial z]^{1/2}$ is the local Brunt–Väisälä frequency. The characteristic length scale *l* is given by

18 $$\begin{aligned} l = \min (\varDelta ,l_s), \end{aligned}$$

with $l_s=c_l\sqrt{k_{\textit{sgs}}}N^{-1}$ a stability related length scale. Finally, the subgrid-scale Prandtl number and the model coefficient $c_\epsilon$ depend on the length scale, i.e.,

19 $$\begin{aligned} \textit{Pr}_{\textit{sgs}}^{-1} = 1+\frac{2l}{\varDelta } \quad \text {and} \quad c_\epsilon =c_{\epsilon 1} + \frac{c_{\epsilon 2}l}{\varDelta }. \end{aligned}$$

For the model coefficients $(c_{\epsilon 1},c_{\epsilon 2},c_l)$ the values proposed by Stevens et al. (2000) are used: (0.225, 0.705, 0.82).

**Wall stress and heat-flux model** At the bottom of the domain, the effect of the wall on the flow is modelled with classic Monin–Obukhov similarity theory (Moeng 1984), i.e., the wall stress $\tau _\mathrm{w}=\rho _0u_*^2$ and heat flux $q_\mathrm{w}=-\theta _*u_*$ are estimated from the velocity $M_1$ and potential temperature $\theta _1$ in the first grid point (at height $z_1$),

20 $$\begin{aligned} u_*= \frac{\kappa M_1}{\displaystyle \ln \left( \frac{z_1}{z_0}\right) -\varPsi _m\left( \frac{z_1}{L}\right) }, \end{aligned}$$

21 $$\begin{aligned} \theta _*= \frac{\kappa \left( \theta _1 - \theta _s\right) }{\displaystyle \ln \left( \frac{z_1}{z_0}\right) -\varPsi _h\left( \frac{z_1}{L}\right) }, \end{aligned}$$

with $\theta _*$ the surface-layer temperature scale, $\kappa$ the von Kármán constant, $z_0$ the aerodynamic roughness length, $\theta _s$ the imposed surface temperature and *L* the Obukhov length, defined as

22 $$\begin{aligned} L = \frac{\theta _0u_*^2}{\kappa g \theta _*}. \end{aligned}$$

Stability effects are accounted for by the correction functions $\varPsi _m$ and $\varPsi _h$, for which we use the classical Businger–Dyer form for moderately stable conditions (Businger et al. 1971):

23 $$\begin{aligned} \varPsi _m(\zeta )= & {} -\beta _m \zeta , \end{aligned}$$

24 $$\begin{aligned} \varPsi _h(\zeta )= & {} -\beta _h \zeta , \end{aligned}$$

with $\beta _m=4.8$ and $\beta _h=7.8$ according to the recommendations of the GABLS1 intercomparison study (Beare et al. 2006). Equations 20 and 21 are implicit expressions for $u_*$ and $\theta _*$ due to the dependence of the stability correction functions on *L*. The simple form of the correction functions (Eqs. 23 and 24) allows to solve these equations analytically (see, e.g., Blümel 2000).

Finally, the estimates of the wall stress and heat flux are added as a body force in the first grid cell adjacent to the wall, thereby assuming that the stress is aligned with the velocity vector:

25a $$\begin{aligned}&\displaystyle \tau _{\mathrm{w}1} = -\rho _0u_*^2\frac{\hat{\tilde{u}}_1}{|\hat{\tilde{u}}|},\end{aligned}$$

25b $$\begin{aligned}&\displaystyle \tau _{\mathrm{w}2} =-\rho _0u_*^2\frac{\hat{\tilde{u}}_2}{|\hat{\tilde{u}}|}, \end{aligned}$$

with the horizontal velocity magnitude $|\hat{\tilde{u}}|= \left( \hat{\tilde{u}}_1^2 + \hat{\tilde{u}}_2^2\right) ^{0.5}$. Locally-averaged horizontal velocities, denoted with a hat, are used to match the average wall stress with the classic log law (Bou-Zeid et al. 2005). A filter width of $4\varDelta$ is used. Note that Eqs. 20–21 are also evaluated using locally averaged values, i.e., $M_1=|\hat{\tilde{u}}|\left( z_1\right)$ and $\theta _1=\hat{\tilde{\theta }}\left( z_1\right)$.

**Actuator disk model** The effect of the wind turbines on the flow is modelled using a non-rotating actuator disk method (ADM) (Jimenez et al. 2007). This method represents the turbines as porous disks exerting a thrust force perpendicular to the rotor plane. ADM avoids full resolution of the wind-turbine blade geometry and is therefore well-suited to study large wind farms (see, e.g., Calaf et al. 2010; Meyers and Meneveau 2010; Goit and Meyers 2015; Allaerts and Meyers 2017). The performance of ADM has been investigated by several authors (Wu and Porté-Agel 2011; Meyers and Meneveau 2013; Martínez-Tossas et al. 2015; Lignarolo et al. 2016), and it is generally accepted that ADM provides an accurate representation of the turbine far wake and the wake mixing.

The actuator disk method calculates the total thrust force of a turbine as

26 $$\begin{aligned} F=\rho _0\frac{1}{2}C_\mathrm{T}^\prime u _\mathrm{d}^2\frac{\pi }{4}D^2, \end{aligned}$$

with $u_\mathrm{d}$ the local disk-averaged and time-filtered velocity perpendicular to the turbine disk. Here, a one-sided exponential time filter is used with a time constant of 5 s following Goit and Meyers (2015) and Goit et al. (2016). Further, *D* is the rotor diameter and $C_\mathrm{T}^\prime$ is the disk-based thrust coefficient, which takes blade design geometry and local flow angles into account. The thrust force is distributed uniformly over the disk area and subsequently filtered onto the LES grid by means of a Gaussian convolution filter leading to $f_i$ in Eq. 11. Details can be found in Meyers and Meneveau (2010) and Goit and Meyers (2015).

## Appendix 2: Derivation of Linear Boundary-Layer Displacement Model

Here, we derive a linear one-dimensional model for the boundary-layer response to a given disturbance. A similar approach has been used by Smith (2007, 2010) to study the perturbation of the boundary layer by small-scale orography or constant drag forcing, representing the effect of a large wind farm. Here, we consider the boundary-layer behaviour in response to a wind-farm drag force that varies linearly with the perturbation velocity.

First, the time- and vertically-averaged Navier–Stokes equations (per unit mass) for a boundary layer with height *h* and mean flow aligned with the *x*-direction can be written as (assuming only variations in the *x*-direction and neglecting Coriolis effects and horizontal diffusion due to turbulence or dispersive stresses)

27 $$\begin{aligned} \frac{\partial Uh}{\partial x}= & {} 0, \end{aligned}$$

28 $$\begin{aligned} U\frac{\partial U}{\partial x}= & {} -\frac{1}{\rho _0}\frac{\partial P}{\partial x}-\frac{F_\mathrm{t}}{h}-\frac{F_\mathrm{d}}{h}, \end{aligned}$$

with *U* and *P* the time- and vertically-averaged velocity and pressure. The friction with the ground is represented by $F_\mathrm{d}=c_\mathrm{d}U^2$, so $c_\mathrm{d}=u_*^2/U^2$. There is no momentum exchange with the free atmosphere by turbulence fluxes as turbulence goes to zero at the boundary-layer top. The term $F_\mathrm{t}$ represents the thrust force exerted by the wind turbines and is calculated as follows. The total thrust force of the wind farm is

29 $$\begin{aligned} F_\mathrm{tot} = \sum _i^{N_\mathrm{t}} \rho _0\frac{1}{2}C_\mathrm{T}^\prime u_{\mathrm{d},i}^2\frac{\pi }{4}D^2 = N_\mathrm{t}\rho _0\frac{1}{2}C_\mathrm{T} \eta _\mathrm{t} u_\mathrm{fs}^2\frac{\pi }{4}D^2, \end{aligned}$$

with $u_{\mathrm{d},i}$ the velocity at the rotor disk of turbine *i*, $C_\mathrm{T}^\prime$ and $C_\mathrm{T}$ the disk-based and conventional thrust coefficient, $\eta _\mathrm{t}$ a wake efficiency based on the thrust force, and $u_\mathrm{fs}$ the free-stream velocity upstream of the first turbine row (i.e., the velocity measured upstream before local pressure build up in front of the turbine slows down the approaching flow). We assume that the wind-farm force is mixed homogeneously between the turbines in the wind-farm area and is zero outside. Thus, dividing Eq. 29 by the total farm area $N_\mathrm{t}s_xs_yD^2$ and the density $\rho _0$ yields the wind-farm thrust force per surface area and per unit mass $F_\mathrm{t}=c_\mathrm{t} U^2 {\Pi }(x)$ with ${\Pi }(x)$ a unit pulse (i.e., equal to one inside the wind farm and zero everywhere else) and

30 $$\begin{aligned} c_\mathrm{t} = \frac{1}{2}\frac{\pi C_\mathrm{T}}{4s_xs_y}\,\eta _\mathrm{t}\,\gamma , \end{aligned}$$

with $\gamma =u_\mathrm{fs}^2/U^2$ a wind-profile shape factor. The wake efficiency $\eta _\mathrm{t}$ based on the thrust force can be related to the wake efficiency based on the power (defined in Eq. 2) by $\eta _\mathrm{t} = \eta _\mathrm{w} u_{\mathrm{d},1}\sum _i^{N_\mathrm{t}}u_{\mathrm{d},i}^2/\sum _i^{N_\mathrm{t}}u_{\mathrm{d},i}^3$. For the sake of simplicity, we take $\eta _\mathrm{t} \approx \eta _\mathrm{w}$. Furthermore, we assume that the shape factor $\gamma$ is only determined by the atmospheric conditions and not affected by the wind farm or the gravity waves, so that it can be computed far upstream of the wind farm (e.g., in Sect. 5.1, we use values from the precursor simulation).

In a second step, the variables (*U*, *P*, *h*) are split into a mean background value ($\overline{U},\overline{P},H$) and a perturbation quantity ($u,p,\eta$). Linearising Eq. 28 around the background state yields

31 $$\begin{aligned} \overline{U}\frac{\partial u}{\partial x}=-\frac{1}{\rho _0}\frac{\partial p}{\partial x}-\frac{c_\mathrm{t}{\Pi }(x)\overline{U}^2}{H}-\frac{2c_\mathrm{t}{\Pi }(x)\overline{U} u}{H}-\frac{2c_\mathrm{d}\overline{U} u}{H}, \end{aligned}$$

where we assume that the background pressure gradient balances the mean surface drag, i.e., $\partial \overline{P}/\partial x = -\rho _0c_\mathrm{d} \overline{U}^2/H$. The pressure perturbation is comprised of two terms (Smith 2010): a first part is caused by the displacement of the inversion layer and is given by $p_1/\rho _0=g^\prime \eta$, with $g^\prime =g\varDelta \theta /\theta _0$ a reduced gravity accounting for the inversion strength; the second part $p_2$ is caused by vertically-propagating gravity waves in the free atmosphere. In Fourier components, the latter is given by $im\hat{p}_2/\rho _0=- N^2 \hat{\eta }$ with $m=\text {sign}(k)N/U_g$ the vertical wavenumber (using the hydrostatic assumption), *N* the Brunt–Väisälä frequency and $U_g$ the *x*-velocity in the free atmosphere. Hence, the derivative with respect to *x* can be written as $ik\hat{p}_2/\rho _0=-|k|NU_g \hat{\eta }$, or in real space,

32 $$\begin{aligned} \frac{1}{\rho _0}\frac{\partial p_2}{\partial x}=-\frac{NU_g}{2\pi }\int _k |k| \left( \int _{x^\prime } \eta \left( x^\prime \right) \hbox {e}^{-ikx^\prime }\,\mathrm {d}x^\prime \right) \hbox {e}^{ikx} \,\mathrm {d}k \equiv -NU_g\mathscr {G}(\eta ), \end{aligned}$$

where we introduced the linear operator $\mathscr {G}$ as a shorthand notation for the double integration operation on $\eta$.

Finally, we use the linearised continuity equation ($\overline{U}\eta + Hu=0$) to transform Eq. 31 into an ordinary differential equation in $\eta$. Multiplying with $-H/\overline{U}^2$ and rearranging the pressure terms yields

33 $$\begin{aligned} \left( 1-\textit{Fr}^{-2}\right) \frac{\partial \eta }{\partial x}= c_\mathrm{t}{\Pi }(x) - 2 \left( c_\mathrm{t}{\Pi }(x)+c_\mathrm{d}\right) \frac{\eta }{H}-P_N^{-1}\mathscr {G}(\eta ), \end{aligned}$$

with $$\textit{Fr}^2=\overline{U}^2/g^\prime H$$ and $$P_N=\overline{U}^2/(NU_gH)$$.
